# Regarding: ‘Costs of managing adverse events in the treatment of first-line metastatic renal cell carcinoma: bevacizumab in combination with interferon-α2a compared with sunitinib’

**DOI:** 10.1038/sj.bjc.6605585

**Published:** 2010-02-23

**Authors:** C Porta

**Affiliations:** 1 Medical Oncology, IRCCS San Matteo University Hospital Foundation, Pavia, Italy

**Sir,**

Mickisch *et al* (2010) report a comparison of the costs of managing side effects due to treatment with either sunitinib or bevacizumab plus interferon, the two presently available standards of care for the first-line treatment of advanced kidney cancer, in four European countries, i.e., Germany, UK, France, and Italy.

This analysis is of value for clinical practice given the recent availability of several molecularly targeted therapies for the treatment of advanced kidney cancer, including bevacizumab and sunitinib, which have radically changed the natural history of the disease. These therapies have made – at least in the vast majority of cases – this once orphan tumour more likely a chronic disease. Such relative long-term disease stabilisation is inevitably accompanied by increasing costs and, even though these new therapies are usually quite well tolerated, side effects may nevertheless occur, which leads to the need for further resources such as extra medical treatment or hospitalisation. As we do not presently have predictive features that could help us to *a priori* select responders to treatments such as bevacizumab and sunitinib, it is important that one should take into account *both* the primary costs of each of these treatments – in some countries artificially made uniform by regulatory authorities – and also secondary costs, such as those related to the management of these therapy-induced side effects.

Mickisch *et al* used pivotal trial data (Escudier *et al*, 2007; Motzer *et al*, 2007) and financial figures obtained from the healthcare systems of each of the four countries; a linear decision analytic model was then used to calculate the cost comparison. The average cost per patient for the management of grade 3 and 4 side effects was markedly higher with sunitinib, as compared with bevacizumab plus interferon. Despite a relevant cost variability among the four countries, sunitinib treatment led to an average increase in the costs of treatment, owing to the management of therapy-related grade 3 and 4 side effects, of 637.5 € (ranging from 418 € for Germany to 972 € for France). Notably, such a difference could also be considered as an underestimation; indeed, we already know that lowering the dose of interferon can lead to a reduction in side effects experienced by patients receiving the bevacizumab–interferon combination, whereas efficacy seems to be maintained (Melichar *et al*, 2008), therefore potentially leading to further savings. These savings are both in terms of a reduction in the amount of interferon used, as well as in the resources necessary for the management of interferon-related side effects.

Moreover, toxicities such as hypothyroidism and cardiotoxicity may have been grossly underestimated in the sunitinib registrative trial (Motzer *et al*, 2007) compared with the levels indicated from other recent studies of sunitinib (Rini *et al*, 2007; Chen, 2009; Torino *et al*, 2009; Di Lorenzo *et al*, 2009), whereas the safety profile of bevacizumab plus interferon does not seem to have changed so much over time. When considering these additional facts, the indirect economic advantage of bevacizumab plus interferon seems to be even more evident and convincing.

In sum, the report by Mickisch *et al* (2010) highlights that when deciding the ideal treatment for each given patient affected by advanced kidney cancer entering our hospitals, we should take into account economic factors (secondary costs) in addition to the efficacy and safety profiles of available treatments.
